# Microstructural Characterization of Al/CNTs Nanocomposites after Cold Rolling

**DOI:** 10.3390/nano13081362

**Published:** 2023-04-14

**Authors:** Íris Carneiro, José V. Fernandes, Sónia Simões

**Affiliations:** 1 DEMM—Department of Metallurgical and Materials Engineering, University of Porto, Rua Dr. Roberto Frias, 4200-465 Porto, Portugal; up201207199@fe.up.pt; 2 LAETA/INEGI—Institute of Science and Innovation in Mechanical and Industrial Engineering, Rua Dr. Roberto Frias, 4200-465 Porto, Portugal; 3 School of Metallurgy and Materials, University of Birmingham, Edgbaston, Birmingham B15 2TT, UK; 4 Centre for Mechanical Engineering, Materials and Processes (CEMMPRE), ARISE, Department of Mechanical Engineering, University of Coimbra, Rua Luís Reis Santos, Pinhal de Marrocos, 3030-788 Coimbra, Portugal

**Keywords:** powder metallurgy, deformation behaviour, cold rolling, metal matrix nanocomposites, carbon nanotubes

## Abstract

The deformation behaviour of aluminium reinforced by carbon nanotubes (Al/CNTs) nanocomposites during cold rolling was investigated in this work. Deformation processes after production by conventional powder metallurgy routes may be an efficient approach to improve the microstructure and mechanical properties by decreasing the porosity. Metal matrix nanocomposites have enormous potential to produce advanced components, mainly in the mobility industry, with powder metallurgy being one of the most reported production processes. For this reason, it is increasingly important to study the deformation behaviour of nanocomposites. In this context, nanocomposites were produced via powder metallurgy. Advanced characterization techniques carried out the microstructural characterization of the as-received powders and produced nanocomposites. The microstructural characterization of the as-received powders and produced nanocomposites was carried out through optical microscopy (OM), and scanning and transmission electron microscopy (SEM and TEM), complemented by electron backscattered diffraction (EBSD). The powder metallurgy route followed by cold rolling is reliable for Al/CNTs nanocomposites. The microstructural characterization shows that the nanocomposites exhibit a different crystallographic orientation than the Al matrix. CNTs in the matrix influence grain rotation during sintering and deformation. Mechanical characterization revealed that during deformation, there is an initial decrease in the hardness and tensile strength for the Al/CNTs and Al matrix. The initial decrease was attributed to the Bauschinger effect being more significant for the nanocomposites. The difference in the mechanical properties of the nanocomposites and Al matrix was attributed to distinct texture evolution during cold rolling.

## 1. Introduction

Growing economic implications and political awareness of the challenge of climate change have driven the research and development of new alternatives, especially in processing advanced materials [1]. Metal matrix nanocomposites (MMNCs), especially lightweight matrices, can be an interesting approach to meet the requirements of reducing CO_2_ emission levels in the mobility industry. Different types of reinforcement are reported for this type of nanocomposites: SiC, TiC, Al_2_O_3_, Si_3_N_4_, and carbon-based nanomaterials such as carbon nanotubes (CNTs) [2,3,4,5,6].

Due to the extraordinary physical and mechanical properties of CNTs [7,8], this is a potential reinforcement for components for the aerospace and automotive industries with aluminium [9,10]. In producing CNT-reinforced MMNCs, several challenges must be overcome to achieve effective reinforcement. These consist of: (1) homogeneous dispersion of the reinforcement in the metal matrix; (2) achieving a solid bond at the matrix/reinforcement interface for effective load transfer; (3) avoiding damage to the reinforcement structure during the dispersion process; and (4) reducing the chemical reaction between the reinforcement and the matrix during the production process [11,12,13].

Classical powder metallurgy (PM), using different kinds of sintering, is one of the most reported processes for successfully producing CNT-reinforced aluminium matrix [12,14,15,16,17,18]. However, the typical porosity of this process can compromise the mechanical properties of components produced by PM [19]. Plastic deformation after PM, such as extrusion [20,21,22,23], forging [24,25], and rolling [26,27,28], can be an option to improve the density of nanocomposites produced by this process. Esawi et al. [20] investigated the mechanical properties of aluminium composites reinforced with CNTs, produced by cold compaction followed by hot extrusion. The mixtures were produced by ball milling. The authors observed that an increase in the mechanical properties of nanocomposites attests to the effect of reinforcement mechanisms. Choi et al. [21] also showed that combining powder metallurgy with deformation processes, such as hot extrusion, effectively produces aluminium reinforced by CNTs (Al/CNTs) nanocomposites. These researchers combine ball milling and hot extrusion techniques to produce nanocomposites that have been shown to be effective in increasing the final samples’ mechanical properties. Hot extrusion has some advantages in producing these nanocomposites due to involving plastic deformation, as it promotes grain size reinforcement and strain hardening combined with an increase in the nanocomposites’ relative density which is positive for obtaining final high mechanical properties.

Deformation processes are not only used to improve the dispersion of the CNTs and the density of the nanocomposites but, as shown by Liu et al. [24], can also be used to align the CNTs and thus achieve a significant increase in mechanical strength. This work uses forging after production by powder metallurgy to obtain better dispersion and a better interface between the CNTs and the Al matrix. The authors observed a significant increase in mechanical properties, which they attributed to a more effective load transfer mechanism.

Rolling is also one of the most reported post-processing processes for application in Al/CNTs produced by powder metallurgy. For example, Yuan et al. [26] showed that hot rolling effectively produces Al/CNTs with a homogeneous distribution of reinforced material. Thus, the nanocomposites exhibited an excellent balance between high strength and ductility attributed to load transfer and grain refinement strengthening mechanisms. Guo et al. [27] reported similar results for Al nanocomposites reinforced with CNTs, produced with plasma spark sintering (SPS), followed by hot rolling. Sadeghi et al. [28] produced Al/CNTs nanocomposites by powder metallurgy following a combination of micro/macro rolling processes. This approach has also proved convenient for obtaining a good strength–ductility relationship for the nanocomposite. Reinforcement mechanisms such as load transfer, increased dislocation density, and grain refinement plays a key role in the process. Yuan et al. [29] produced samples by flake powder metallurgy, followed by hot-rolling at 500 °C with ~85% of reduction. This process leads to a lamellar microstructure with uniformly dispersed CNTs and some Al_4_C_3_ reaction products. It is an effective production route.

The combination of powder metallurgy routes with deformation processes can also be applied when using alloys as matrices, as reported by Fan et al. [30]. The authors used cold rolling as a post-sintering technique to investigate the production of 6061Al/CNTs nanocomposite by flake powder metallurgy. The objective was to reduce the grain size of the sample, and promote and evaluate its superplastic deformation behaviour. Before rolling, the powders were mixed using high-energy ball milling, sintered, and hot-extruded. Initially, the rolling was performed at 200 °C for preliminary thickness reduction and then at room temperature. The cold rolling method reduced the grain size from 580 nm to 300 nm, promoting its homogeneity. This potentiated a significant improvement in the mechanical properties of the nanocomposite at high temperatures.

Although cold rolling is a useful post-sintering process that can improve nanocomposites’ microstructure and mechanical properties, the influence of CNTs on the deformation behaviour still needs to be better understood. In this context, this work has the innovation of investigating the deformation behaviour of nanocomposites when subjected to cold rolling. The previously published work [15,16,31] presents the powder metallurgy processing conditions, dispersion methods, and mechanical characterization of Al/CNTs as the main strengthening mechanisms. However, the deformation behaviour still needs to be investigated. The nanocomposites were produced by powder metallurgy followed by cold rolling to achieve this goal. Microstructural and mechanical characterization was performed to investigate the effect of CNTs on the deformation behaviour of the nanocomposites. Hence, it was possible to establish a relationship between the microstructure and the mechanical properties to determine the deformation behaviour of the nanocomposites and the effect of CNTs.

## 2. Materials and Methods

The Al powders were obtained from Goodfellow Cambridge Ltd. (Huntingdon, UK) and the CNTs used were multi-walled from Fibermax Nanocomposites Ltd. (London, UK). The parameter D50, indicating the size below which 50% of the measured Al particles are found, was measured using dynamic light scattering (DLS) and exhibited a value of 32 μm [31]. The morphology and microstructure of the powders were evaluated using optical microscopy (OM), scanning electron microscopy (SEM), and electron backscatter diffraction (EBSD). The equipment used for this characterization was an optical microscope, M 4000 M, with Leica Application Suite software (version 4.13.0, Leica Microsystems, Wetzlar, Germany) and a Thermo Fisher Scientific QUANTA 400 FEG SEM (Thermo Fisher Scientific, Hillsboro, OR, USA) with an EBSD TSL-EDAX detector unit (EDAX Inc. (Ametek), Mahwah, NJ, USA).

**Figure 1:**
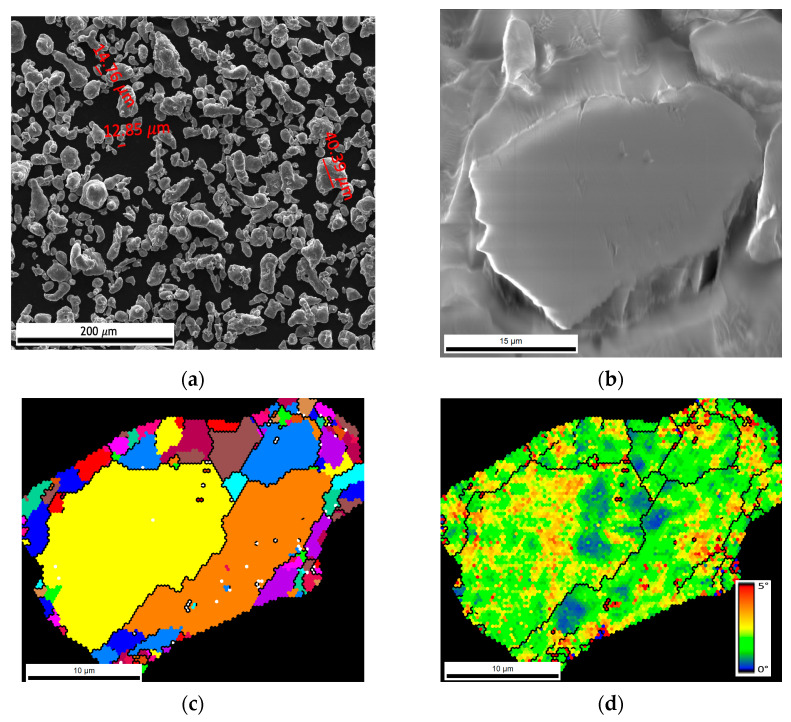
(**a**) Scanning electron microscopy (SEM) image of Al powder, (**b**) higher magnification of SEM image showing an Al particle, (**c**) unique grain colour map showing that more than one grain is present in the Al particle, where each colour represents a different grain, and (**d**) Kernel average misorientation (KAM) map.

**Figure 2:**
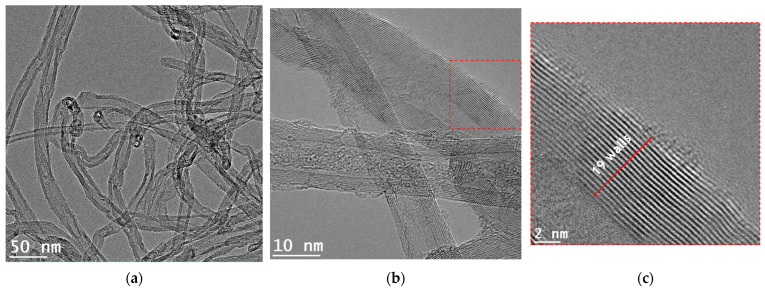
Transmission electron microscopy (TEM) and high-resolution TEM (HRTEM) images of CNTs. (**a**) TEM image of dispersed CNTs, (**b**) HRTEM of the CNTs, and (**c**) a HRTEM detail showing the number of walls in a CNT.

Figure 1 shows the SEM image and EBSD characterization of the as-received Al powders. The powders have an almost spherical morphology, although some particles have an elongated shape. Based on the EBSD analysis, the Al particles have more than one grain and are deformed (Figure 1d). In Kernel average misorientation (KAM) maps, a high average misorientation value is visible across the particle. This can have occurred during the production process, as similar results were observed in previous works for nickel powders [32]. Figure 2 shows the CNTs images observed in transmission electron microscopy (TEM, FEI Company, Hillsboro, OR, USA) and high-resolution TEM (HRTEM, JEOL Ltd., Tokyo, Japan) that exhibit a bamboo-like structure, and 19 walls can be measured in the nanotube shown.

The nanocomposites were produced by conventional powder metallurgy routes. Al powders and CNTs were dispersed and mixed by ultrasonication in isopropanol for 15 min using an IKA T25 digital ULTRA-TURRAX (Staufen im Breisgau, Baden-Wuerttemberg, Germany) disperser with 20.4 KHz. The isopropanol was filtered, and the powder mixtures were dried and cold pressed at 300 MPa into discs of around 6 mm diameter and 2 mm thickness. Sintering was performed in a horizontal tubular furnace, under a vacuum lower than 10^−3^ Pa, at 640 °C for 90 min. This procedure is described in detail in previous works [15,16,31]. The deformation behaviour was investigated during cold rolling. Nanocomposites Al/CNTs and Al matrix samples produced under the same conditions were rolled at room temperature, with low rotation speed (around 10 rpm) and with an average of 4 passes up to different strain values (0.11, 0.36, and 0.69).

**Figure 3:**
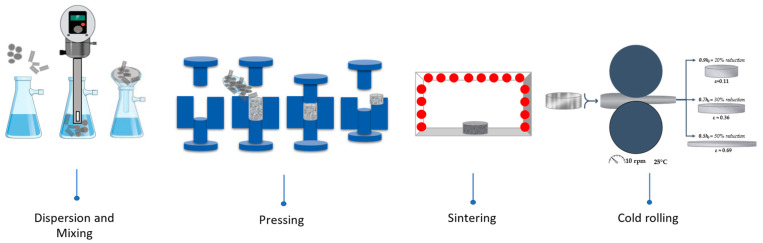
Schematic representation of the production steps used in Al matrix and Al/CNTs nanocomposites.

Figure 3 shows a schematically illustrated sequence from the dispersion/mixture step of the powders and CNTs to the cold rolling process. The microstructural characterization of the nanocomposites was performed by SEM and EBSD analysis. The EBSD data were analysed by TSL OIM Analysis 5.2 (Ametek Inc., Devon-Berwyn, PA, USA) and the ATEX version 3.28 (University of Lorraine, Metz, France) [33].

The mechanical properties were studied using microhardness Vickers tests (98 mN for 15 s) to evaluate the hardness evolution. These tests were performed on a Duramin-1 equipment (Struers, Ballerup, Denmark). The tensile test was performed with a velocity of 1 mm/s, using Shimadzu EZ Test equipment (Shimadzu Corporation, Kyoto, Japan), and four samples of each, nanocomposite and aluminium matrix, were tested.

## 3. Results and Discussion

**Figure 4:**
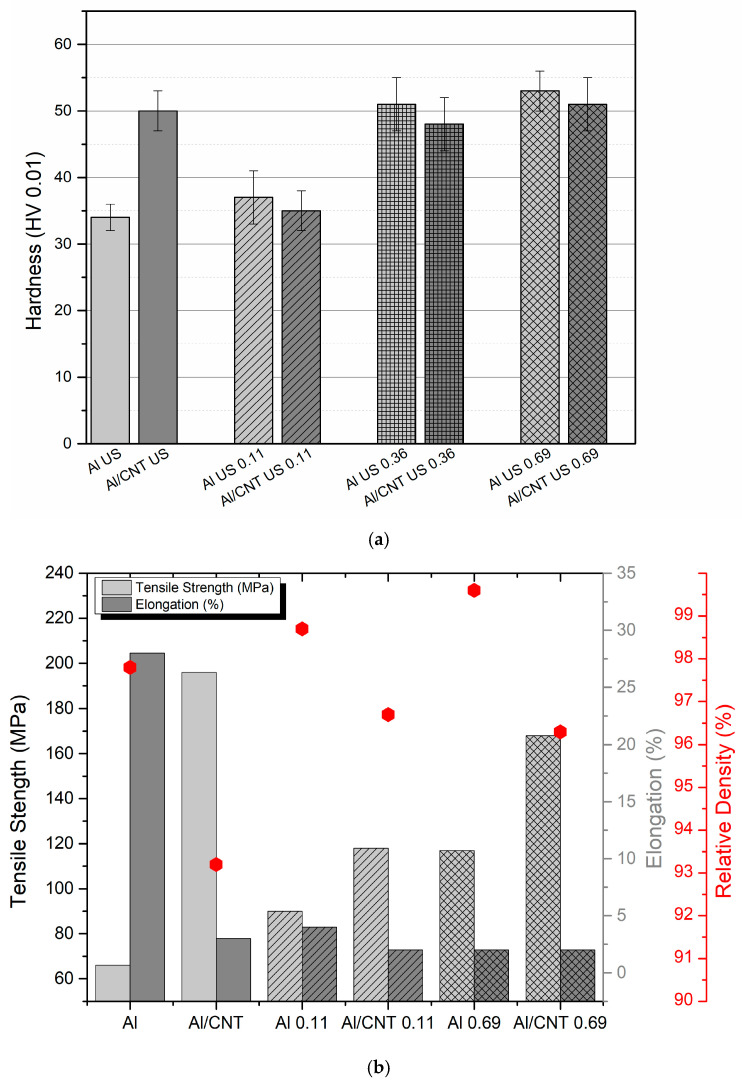
(**a**) Vickers microhardness and (**b**) tensile strength, elongation, and density for the Al/CNTs nanocomposites and Al matrix as-sintered and cold rolled up to different strain values. The different column grid represents different applied strains.

The mechanical properties of the sintered nanocomposites and Al matrix subjected to cold rolling were evaluated to investigate the deformation behaviour. Figure 4 shows the hardness, tensile strength, elongation, and density of the sintered samples and those laminated with different strains. The hardness results show a decrease for the lowest strain value of 0.11 for the Al matrix and Al/CNTs nanocomposites. For the other strain values, higher hardness values are observed for the Al matrix than for the nanocomposite. For the nanocomposites, the hardness for strains of 0.36 and 0.69 was approximately equal to the initial strain (sintered sample) and similar to the Al matrix. Previous work on Ni/CNTs nanocomposites [34] revealed that the nanocomposites and Ni matrix exhibit softening for low strain values of 0.11 and 0.22. However, for higher strains (0.36), an increase in hardness was already observed for the Ni/CNT nanocomposites concerning the sintered sample, although the nanocomposites exhibited a lower value than the Ni matrix submitted to cold rolling under the same conditions. The relative density values demonstrate that cold rolling effectively eliminates the porosity of the sintered samples. However, a decrease was observed for the cold rolling nanocomposites with a rate of 0.69. This decrease can be attributed to pores or microcracking that can form for this condition due to the brittleness of these nanocomposites. The tensile strength values for the lower strain (0.11) follow the hardness evolution. A significant decrease occurs for both samples. There was a decrease in the tensile strength relative to the sintered nanocomposites for higher strain, but the value showed higher mechanical strength than the Al matrix. For comparison purposes, the microstructural characterization of the Al/CNTs nanocomposites and the Al matrix produced under the same conditions was performed to understand the hardness evolution with the nanocomposites’ deformation.

**Figure 5:**
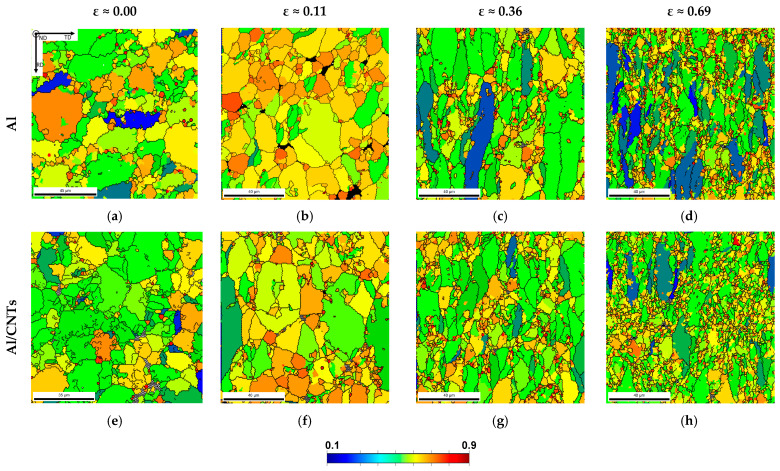
Grain shape aspect ratio maps of (**a**–**d**) Al and (**e**–**h**) Al/CNTs samples, cold rolled to strains of 0.00, 0.11, 0.36, and 0.69.

The microstructural characterization of the samples with plastic deformation was performed to establish a relationship with the mechanical properties of the nanocomposites. The grain shape aspect ratio of the samples was evaluated to study whether the composites behaved differently from the aluminium matrix. Figure 5 shows the samples’ grain shape aspect ratio maps with increasing strains.

The red grains are equiaxed, while the blue grains are elongated in shape. With increasing strain, it is evident that the grain shape changes from equiaxial to elongated in the rolling direction. This change promotes a decrease in the aspect ratio. The change is less evident for the nanocomposites compared to the Al matrix. For the highest strains, the elongated grains appear to have a smaller size in the nanocomposites. This can be explained due to the fragmentation of the grains after being significantly elongated in the rolling direction, leading to a smaller decrease in the width/height ratio of the grains in the case of the nanocomposite. Due to this fragmentation effect, the grain aspect ratio values decrease with deformation and are less significant for the nanocomposites than the aluminium matrix. The difference in aspect ratio between the Al and nanocomposites shows that the deformation behaviour is not occurring similarly for both samples. The nanocomposites have higher initial mechanical properties (hardness and tensile strength), so the work hardening is less than the Al matrix. Therefore, the cold-rolled nanocomposite samples present fewer elongated grains than the Al matrix. This can be explained since the presence of CNTs mainly at the grain boundaries acts as an obstacle to the movement of dislocations, which can explain more fractured and less elongated grains in these samples. These results help to understand why the hardness of the samples with plastic deformation is equivalent. The nanocomposites present lower hardness than expected due to their inherent brittleness or tendency to crack under strain. This can be explained due to the work-hardening difference between the nanocomposite and the Al matrix. Other authors reported similar results studying the deformation during the rolling of samples produced by powder metallurgy [35].

Regarding grain size, the cold rolling process did not affect the average value. The average grain size of the sintered and cold-rolled samples is shown in Table 1. Sintered and rolled samples show similar values for the average grain size value, ranging from 13.2 and 12.1 μm for sintered Al and Al/CNTs samples to 12.8 and 12.0 μm for cold rolled samples at 0.69 for Al and Al/CNTs.

**Figure 6:**
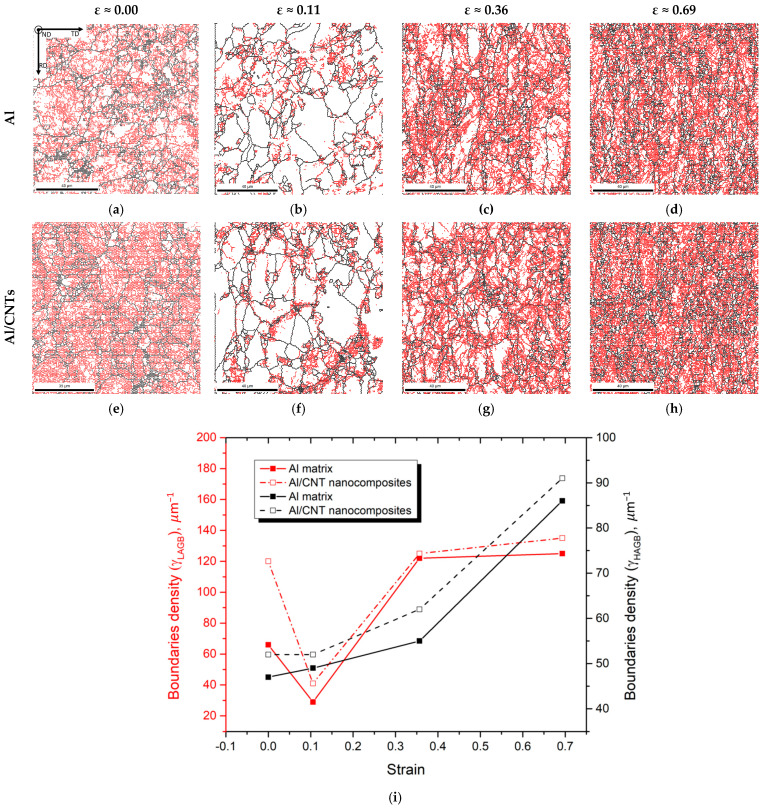
Electron backscatter diffraction (EBSD) maps showing the different boundary types (black—HAGB and red—LAGB). (**a**–**d**) Al and (**e**–**h**) Al/CNT samples, cold rolled to strains of 0.00, 0.11, 0.36, and 0.69, and (**i**) evolution of LAGB and HAGB for the Al and Al/CNTs samples, as-sintered and cold rolled.

The character of the grain boundaries was another microstructural parameter evaluated to study the deformation behaviour of the nanocomposites. Figure 6 shows the grain boundary maps and the grain boundary misorientation distribution for the samples of cold-rolled Al and Al/CNTs under different strains. In the grain boundary maps, the high-angle grain boundaries (HAGB) distribution is marked in black, and the low-angle grain boundaries (LAGB) are red. A high fraction of LAGB characterizes the Al and Al/CNT microstructures for the higher strains. However, few differences were detected between the Al and Al/CNTs samples. For the lower strains, few LAGBs were present in the samples. When comparing these values with the values of the sintered samples, the LAGB values of the samples deformed to 0.11 are lower, especially for the nanocomposites. This means that there was a decrease in these boundaries with plastic deformation. The HAGB fraction increases for the 0.36 and 0.69 strains, which is slightly sharper for the nanocomposites. This may be associated with grain fragmentation, which is more significant for the nanocomposites due to the presence of CNTs. Similar results were observed for lower strains in previous work on the deformation behaviour of Ni and Ni/CNTs samples [34] produced by powder metallurgy and submitted to cold rolling. In these cases, for strains of 0.11 and 0.22, a significant decrease in LAGB was observed, mainly for the nanocomposites. Furthermore, for strains greater than 0.36, the LAGB increases; consequently, the HAGB and the coincidence site lattice (CSL) boundaries decrease. Similarly, the Al/CNT and Ni/CNT nanocomposites show a higher fraction of LAGB than the respective matrix when there is an increase in cold rolling deformation.

**Figure 7:**
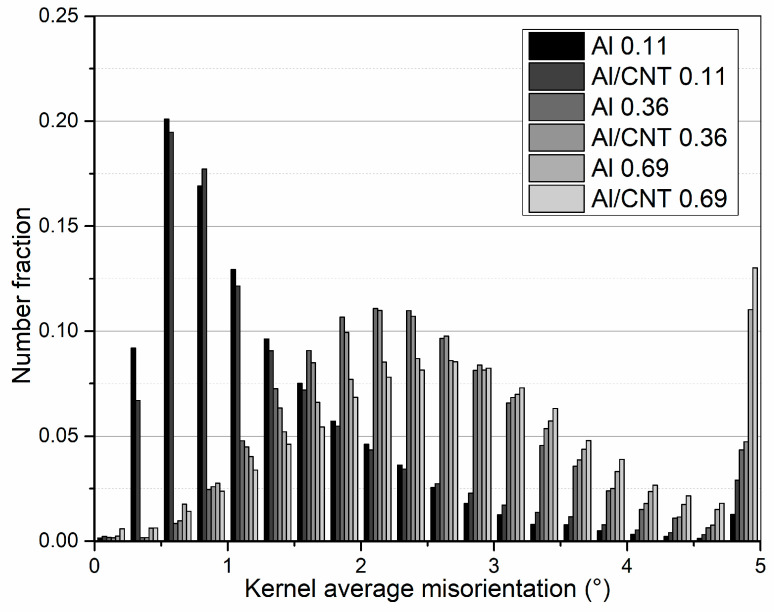
Distribution of Kernel average misorientation (KAM) for Al and Al/CNT samples, cold rolled to strains of 0.11, 0.36, and 0.69.

Figure 7 shows the Kernel average misorientation (KAM) distribution for the Al and Al/CNT cold-rolled samples. From the graph, it is clear that in the cold-rolled samples with the lowest strain (0.11) a lower misorientation angle distribution was observed. This means these samples exhibited a less deformed microstructure, i.e., a lower misorientation average angle. On the contrary, the samples deformed with the highest strain rate (0.69) show a distribution with a wider range of misorientation angles, increasing the misorientation average angle (KAM). The increase in the sample’s plastic deformation can explain the observed results. The average KAM angle increases with increasing strain. A greater sample misorientation was observed for the samples cold rolled with 0.36 and 0.69, in agreement with the increased LAGB fraction already mentioned.

**Figure 8:**
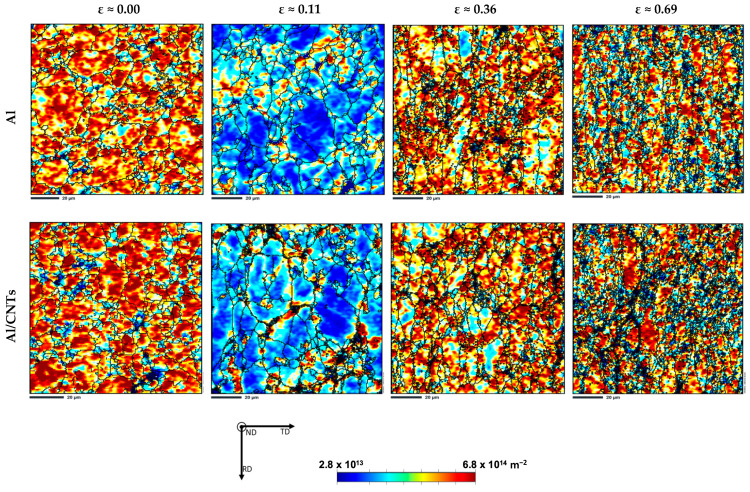
Estimated average geometric necessary dislocation (GNDs) density maps of Al and Al/CNTs samples, sintered and cold rolled to 0.11, 0.36, and 0.69.

**Figure 9:**
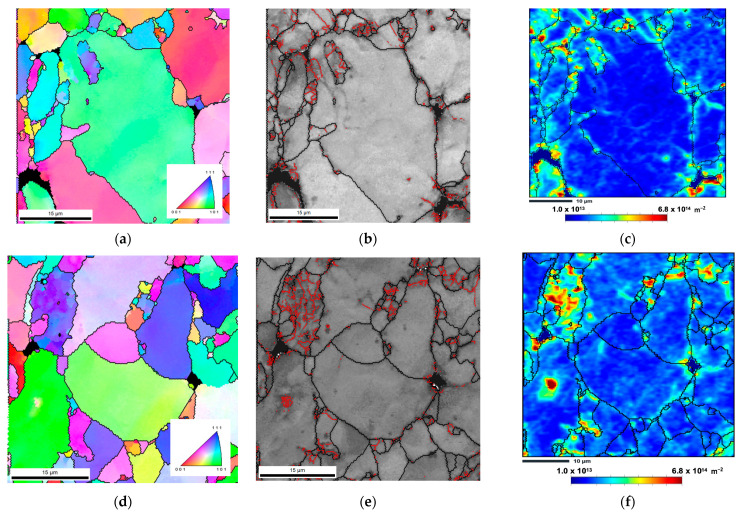
(**a**,**d**) Inverse pole figure (IPF) maps, (**b**,**e**) grain boundaries maps with LAGB delineated in red, and (**c**,**f**) estimated GNDs maps of Al and Al/CNT samples, cold rolled to 0.11.

To investigate the presence of dislocations and correlate them with LAGB and HAGB values, distribution maps of estimated geometrically necessary dislocations (GNDs) were performed. Figure 8 shows these maps and reveals that the GNDs are located mainly at the grain boundaries, indicating a decrease in dislocation density after cold rolling up to 0.11. The average values of the estimated dislocation density evolved from the sintered to the deformed samples up to 0.11, from 1.6 × 10^14^ to 9.3 × 10^13^ m^−2^ in the Al sample, and from 2.0 × 10^14^ to 1.2 × 10^14^ m^−2^ in the nanocomposites. This decrease can be attributed to the Bauschinger effect. This phenomenon occurs when dislocations are reorganized and annihilated during strain path reversal, which can occur during rolling deformation after sintering. Higher magnification maps were plotted to understand the Bauschinger effect and the impact on low-strain (0.11) microstructures in the matrix and the nanocomposite. In this sense, Figure 9 shows inverse pole figure (IPF) maps, grain boundaries (with HAGB and LAGB delineated in black and red, respectively), and GNDs maps of Al and Al/CNTs samples cold rolled up to a strain of 0.11.

In these maps, the grains are primarily free of dislocations, and when these are observed, they are organized close to the grain boundaries or in dislocation cells. This means these microstructures were more organized than in the sintered samples, which showed greater misorientations and dislocation densities [31]. The decrease in LAGB is more evident in the nanocomposite than in the Al matrix due to the back stress associated with the presence of CNTs that assists the inversion of dislocation movement, although it happens in both samples. The Bauschinger effect was only observed for strains up to 0.11, and from then on, the GNDs gradually increased with deformation. However, the dislocation density for the larger strain (0.69) seems to stagnate in both the Al matrix and nanocomposite samples. The Bauschinger effect can explain the decrease in the mechanical properties of the samples. For lower strains, this can be understood by the change observed in the microstructure. The reduction in dislocation density, especially for the nanocomposites, promotes a decrease in hardness and tensile strength than the value of the sintered samples. Some authors [36,37,38,39] have already reported this effect in different deformation conditions, such as Xu et al. [36], who analysed tensile-compression tests of Al/CNTs nanocomposites. Chen et al. [37] investigated 6061 aluminium alloy reinforced by CNTs, performing loading–unloading–reloading cyclic tensile tests. Sadeghi et al. [38,39] studied the deformation of Al/CNTs nanocomposites and supported their results in simulation results.

**Figure 10:**
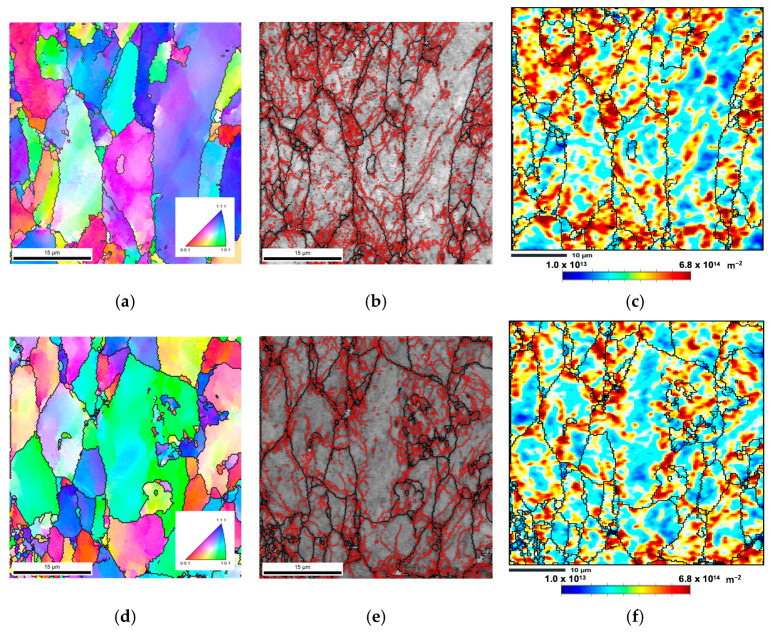
(**a**,**d**) Inverse pole figure (IPF) maps, (**b**,**e**) grain boundaries maps with LAGB delineated in red, and (**c**,**f**) estimated GNDs maps of Al and Al/CNT samples, cold rolled to 0.36.

Furthermore, these authors also mention that the presence of CNTs can influence the Bauschinger effect, occurring during the deformation of the nanocomposite after sintering. However, it is worth noting that the deformation processes applied in these works differ from the cold rolling used in the present work. These studies cannot be used as guidelines, although they are very helpful in understanding the impact of CNTs on this phenomenon. Increasing the strain above 0.36 does not significantly change the LAGB fraction and slightly increases the HAGB for the nanocomposites. This increase can be explained due to the transformation of some LAGB into HAGB. A more significant increase in the LAGB and HAGB fractions was observed for the Al matrix. This is because, in these samples, the rearrangement and multiplication of the LAGB occur more extensively than in the nanocomposites. Due to the presence of the CNTs, this process will be affected in the nanocomposites. Figure 10 shows the IPF maps, grain boundaries, and GNDs maps for samples submitted to rolling at a strain of 0.36.

It is seen that the samples are characterized by a high fraction of LAGB, mainly in the smaller grains. In larger grains, these boundaries are concentrated near the grain boundaries. A large fraction of LAGB is associated with a high density of GNDs. However, the as-sintered nanocomposite samples have already been characterized as having a high LAGB density [31]. This could mean that, even for this strain (0.36), the Bauschinger effect can occur in nanocomposites, which prevents them from increasing LAGB, hardness, and tensile strength. Some smaller grains in these graphs may be associated with HAGB formation due to the accumulation of dislocations during the rolling process. This result can justify the slight increase observed in the fraction of these boundaries. The nanocomposites show superior mechanical properties to the Al matrix, with plastic deformation, but a more significant increase would be expected. This may be associated with some dislocation structure organization due to the Bauschinger effect or damage in the structure CNTs during the deformation process, as reported by some authors [40].

In the initial stages of cold rolling, the texture of the material may be relatively random or isotropic. However, as the material is plastic deformed, the crystal lattice begins to align along the rolling direction, developing a preferred orientation. The specific texture evolution during cold rolling depends on various factors, including the initial crystallographic texture of the material, the degree of deformation, the rolling direction, and the processing conditions. The texture evolution of the Al and Al/CNTs samples was investigated to understand the deformation behaviour of the nanocomposites. Different factors affect the texture evolution during deformation, one of them being the initial crystallographic orientation of the samples.

**Figure 11:**
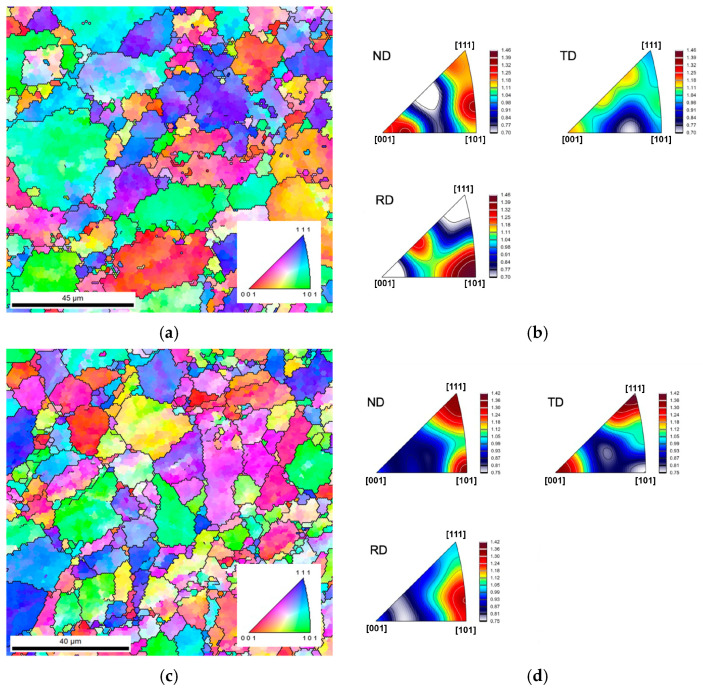
(**a**,**c**) IPF maps and (**b**,**d**) IPF of the sintered Al (**above**) and Al/CNTs (**below**) samples.

For this reason, the initial texture of Al and Al/CNTs as-sintered was evaluated. Figure 11 shows the IPF and IPF maps of the sintered Al and Al/CNTs samples. These results indicate that the addition of CNTs affects the crystallographic grain orientation of the sintered sample, although there is no strong preferred crystallographic orientation. The nanocomposites exhibited a different texture than the samples without reinforcement. For example, concerning the normal (ND) to the plane parallel to the sample surface, the preferred orientations are the [111] and [101] axes for Al/CNTs, which is not the case for the Al matrix (preferred orientation for ND are close to [104] and [313]). The difference can be attributed to the CNT agglomerates in the composites that affect grain orientation and growth during sintering. In previous works, Ni/CNT nanocomposites produced by the same powder metallurgy route [15,34] also exhibited differences in crystallography orientation compared to unreinforced samples.

**Figure 12:**
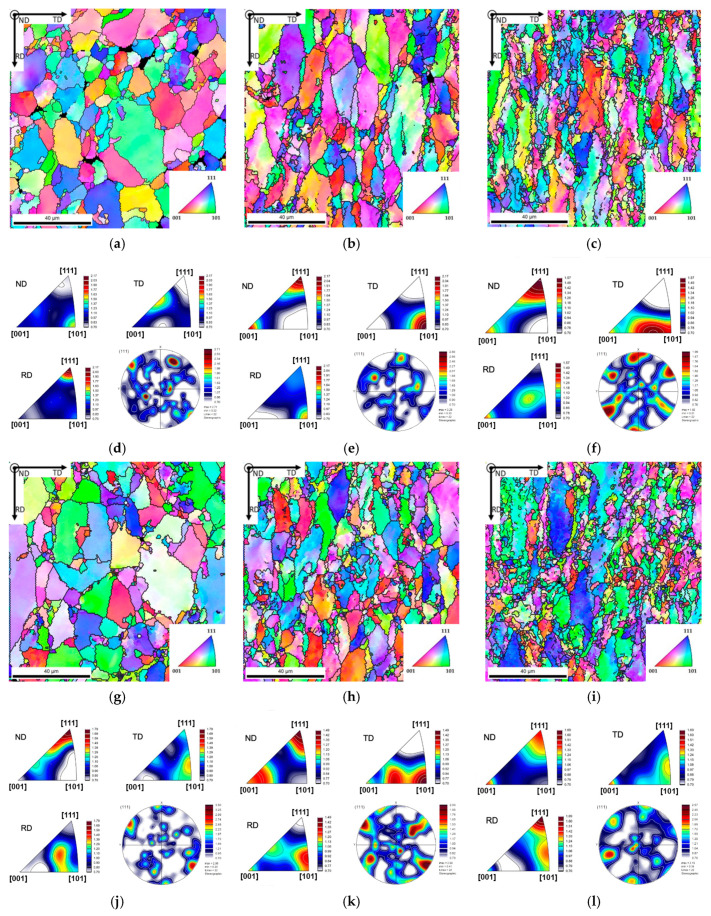

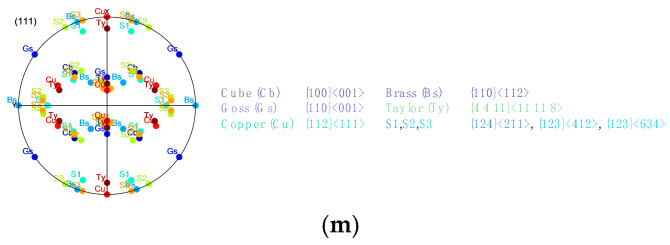
(**a**–**c**;**g**–**i**) IPF maps and (**d**–**f**,**j**–**l**) IPF and [111] PF of the cold rolled (**a**–**f**) Al and (**g**–**l**) Al/CNT samples, deformed with strains of (**a**,**d**,**g**,**j**) 0.11, (**d**,**e**,**h**,**k**) 0.36, and (**c**,**f**,**I**,**l**) 0.69. (**m**) Identification of rolling texture components in [111] pole figure.

During cold rolling, the texture was also evaluated. Figure 12 shows the IPF maps and IPF of the Al matrix and the nanocomposites, cold rolled at 0.11, 0.36, and 0.69. Based on the IPF maps, the crystallographic orientation of the two samples is different during the deformation, mainly because the initial orientation is distinct, as shown previously. However, with increasing rolling deformation, the orientation of the elongated grains tends, among others, towards the same preferential orientation, such that DN is close to [111] in both samples. The Al sample deformed with ε = 0.11 presents a higher fraction Cube component, while the nanocomposite presents Brass. With the strain increase to 0.36, the Goss component has a higher fraction in the Al sample, while the nanocomposite presents mostly Taylor. In the samples deformed with 0.69, the main texture components of Al are Copper and Taylor. For the nanocomposite, the main component observed was Cube.

**Figure 13:**
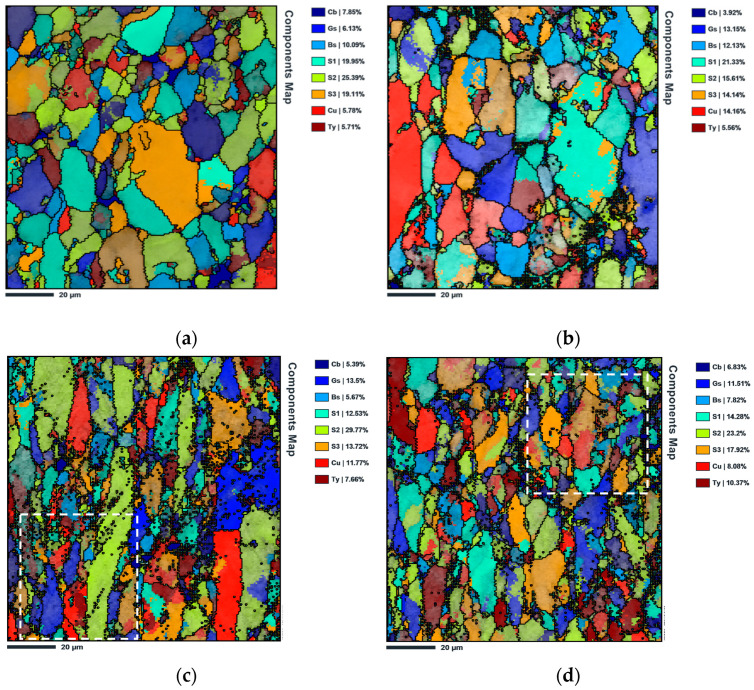

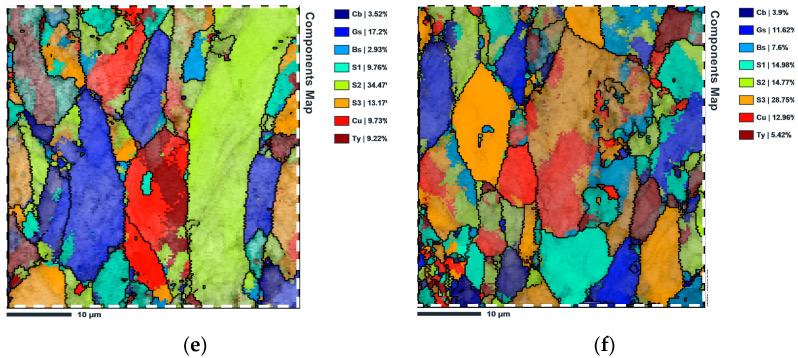
Texture rolling components maps of (**a**) Al cold rolled to 0.11, (**b**) Al/CNT cold rolled to 0.11, (**c**,**e**) Al cold rolled to 0.36, and (**d**,**f**) Al/CNT cold rolled to 0.36 analysed with (**c**,**d**) lower and (**e**,**f**) higher magnification.

Figure 13 shows the maps of the texture components for the Al matrix and Al/CNT nanocomposites, cold rolled up to strains of 0.11 and 0.36. By definition, this type of map represents the orientation of each grain accordingly to the characteristic rolling texture components, accompanied by their volume fraction in each map. It can be seen that for the samples rolled to 0.11, the texture components are different for the two samples. For the strain of 0.36, the samples present similar percentages of the same components showing a high fraction of S2. Thus, CNTs affect texture evolution that is significantly different for small strains. For greater strains, the nanocomposite, although presenting small differences, begins to present a texture that approaches the matrix.

The texture evolution during cold rolling can have important implications for the mechanical properties of the materials. Different texture evolution means that different slip activation during deformation occurs. It is important to note that its texture components do not solely determine the mechanical properties of a material. Other microstructural features such as grain size, defects, and dislocation density can also significantly determine a mechanical behaviour of materials. However, understanding the texture components of a material can provide valuable insights into its potential mechanical properties, which can be useful for designing and engineering materials for specific applications.

The differences in microstructural and texture evolution observed in the sintered Al and Al/CNTs samples may explain the different deformation behaviour of these nanocomposites when subjected to cold rolling. The nanocomposites exhibit a similar hardness to the Al matrix but a higher mechanical strength. This can be explained by heterogeneous deformation as observed in previous work on Cu/CNT [41]; hence, the average hardness is similar for the two samples. However, one would expect a higher tensile strength for the nanocomposites with increasing plastic deformation. The fact that the Bauschinger effect occurs for the lower strain (0.11) may contribute to the sample’s softening during the plastic deformation. Thus, this would be one of the possibilities of the mechanical strength being lower than expected. Microstructural characterization also showed that the dislocation density is similar for cold rolled samples up to strains of 0.36 and 0.69. For the nanocomposites, the high dislocation density in the sintered samples has been shown in previous work [31] to be one of the mechanisms contributing to the strengthening of the nanocomposites and the load transfer mechanism. The annihilation and organization of dislocations during deformation may impair the strengthening effect of these nanocomposites, which may explain the results obtained regarding the mechanical properties. In addition, it is necessary to consider that the deformation process may impair the reinforcement potential since the cold rolling process causes significant damage to the structure of CNTs. Therefore, the damage to the structure of the CNTs may explain the results obtained for mechanical properties.

This work demonstrated that studying the deformation behaviour of nanocomposites is crucial for their implementation. In Al/CNTs nanocomposites, production by powder metallurgy followed by cold rolling proved critical since the deformation behaviour of the matrix is influenced by the reinforcement. The mechanical properties are significantly affected due to the different texture evolution of the nanocomposites.

## 4. Conclusions

Using ultrasonication as a dispersion/mixing technique, the powder metallurgy route efficiently produced Al/CNTs nanocomposites. Cold rolling to different strains allowed us to understand the influence of CNTs on important microstructural features, such as dislocation density, low- and high-angle boundaries, and crystallographic orientation, which affect their mechanical properties under plastic deformation.

The addition of reinforcement successfully improved the Al matrix’s microhardness and tensile strength after sintering. However, the mechanical properties decreased by applying a strain of 0.11 by cold rolling. This reduction resulted from the Bauschinger effect, which promoted the rearrangement and annihilation of dislocations of both samples, Al and Al/CNTs. However, this effect was more substantial in nanocomposites since the reduction in the estimated GND density was more pronounced due to the presence of CNTs.

With increasing strains, the microhardness and tensile strength of the aluminium matrix increased due to a higher fraction of deformed grains. In this sample, the multiplication of dislocations was followed by the formation of high-angle boundaries. On the other hand, no significant increase in hardness was observed for the nanocomposite, being close to those on the Al matrix. Besides that, the Bauschinger effect occurs for applying 0.11 strains, leading to annihilation and rearrangement of dislocations. For the highest strains (0.36 and 0.69), Al and Al/CNTs samples showed similar misorientation angles and dislocation density. However, some deviation in their microhardness values could be associated with slight variations in crystallographic orientation.

The texture evolution during cold rolling showed that the samples presented a different behaviour up to the strain of 0.11. The nanocomposites began to approach the Al matrix with increasing strain. This distinct behaviour can be responsible for the slight differences in hardness between the nanocomposites, and the Al matrix cold rolled at strains of 0.36 and 0.69. The difference in texture evolution can be explained by the difference in crystallographic orientation observed in the sintered samples due to CNTs in the matrix.

## Figures and Tables

**Table 1:** The average grain size of Al and Al/CNTs for sintered and cold-rolled samples.

Average Grain Size (μm)				
	ε = 0.00	ε = 0.11	ε = 0.36	ε = 0.69
Al	13.2 ± 5.8	13.2 ± 6.1	12.5 ± 5.1	12.8 ± 5.6
Al/CNTs	12.1 ± 5.1	12.8 ± 4.7	13.2 ± 6.0	12.0 ± 4.7

## Data Availability

Data can be available upon request from the authors.
